# A Holistic Approach to Cardiometabolic and Infectious Health in the General Population of Reunion Island: The REUNION Study

**DOI:** 10.1007/s44197-024-00221-9

**Published:** 2024-04-02

**Authors:** Mohammad Ryadh Pokeerbux, Patrick Mavingui, Patrick Gérardin, Nelly Agrinier, Erick Gokalsing, Olivier Meilhac, Maxime Cournot

**Affiliations:** 1 Université de La Réunion, UMR Diabète Athérothrombose Réunion Océan Indien (DéTROI), INSERM U1188, Saint-Pierre, La Réunion, 97410 France; 2 Université de La Réunion, UMR Processus Infectieux en Milieu Insulaire et Tropical (PIMIT), CNRS 9192, INSERM U1187, IRD 249, Sainte-Clotilde, La Réunion, 97490 France; 3 Plateforme de Recherche Clinique et Translationnelle, INSERM CIC1410, CHU de La Réunion, Saint-Pierre, La Réunion, 97400 France; 4 CHRU-Nancy, Université de Lorraine, CIC, Epidémiologie clinique, Inserm, Nancy, F-54000 France; 5 Université de Lorraine, Inserm, INSPIIRE, Nancy, F-54000 France; 6 Etablissement Public de Santé Mentale de La Réunion, 42 chemin du Grand Pourpier, 97866 Saint-Paul Cedex, France; 7 Laboratoire IRISSE (IngéniéRIe de la Santé, du Sport et de l’Environnement), Université de La Réunion, UFR SHE, Saint Pierre, EA 4075 France; 8 Groupe de santé Clinifutur, Clinique Les Orchidées, Le Port, La Réunion, 97420 France

**Keywords:** Cardiometabolic Diseases, Infectious Diseases, Reunion Island, Prevalence, Cardiovascular risk Factors, Diabetes, Hypertension, Lipid Profile, Microbiota, Dengue, Chikungunya, Biobank

## Abstract

**Introduction:**

Reunion Island is a French overseas department in the South West Indian Ocean with a unique multi-ethnic population. Cardiovascular diseases are the most common chronic conditions with higher prevalences of hypertension and diabetes compared to mainland France. Moreover, Reunion Island is particularly exposed to vector-borne diseases such as chikungunya and dengue. Our objective is to describe the prevalence of cardiometabolic and infectious diseases in Reunion Island and explore causal mechanisms linking these diseases.

**Methods:**

The REUNION study is an ongoing French prospective study. From January 2022, 2,000 consenting participants (18–68 years old) are being recruited from the general population according to polling lists and random generation of cellphone number. Baseline examination consists of (i) general health examination, assessment of cardiovascular risk factors, markers of subclinical atherosclerosis, bronchial obstruction, neuropathic and autonomic dysfunction, (ii) questionnaires to determine sociodemographic characteristics, diet, exposure to vector-borne diseases, mental health and cognitive functions, social inequalities in health and ethnic origins, (iii) biological sampling for determination of cardiovascular risk factors, seroprevalence of infectious diseases, innovative lipid biomarkers, advanced omics, composition of intestinal, periodontal and skin microbiota, and biobanking.

**Conclusions:**

The REUNION study should provide new insights into the prevalence of cardiometabolic and infectious diseases, as well as their potential associations through the examination of various environmental pathways and a wide range of health aspects.

## Introduction

### Background

Reunion Island is a French overseas department in the Indian Ocean, 700 km east of Madagascar and 140 km west of Mauritius. The island was settled in the 15th century, resulting in mixed population of African, Indian, Chinese, and European descents. Reunion Island benefits from the French universal healthcare system, which is mainly financed by national health insurance and ensures a high standard of care regardless of patient’s income, similar to that of mainland France. Reunion Island is currently experiencing an unprecedented epidemiological transition. With a population of 863,100 according to the latest census on January 1st, 2020, it has one of the fastest aging populations in the world. This transition is characterized by rapid urbanization and socio-economic changes, including an increase in sedentary lifestyles, weight gain, and the consumption of alcohol and salt [1, 2].

Cardiovascular diseases (CVD) are the most common chronic conditions in Reunion Island. The incidences of cerebrovascular disease and ischemic heart disease are far much higher in Reunion Island than in mainland France. The burden of CVD has more than doubled between 2000 and 2009 (+ 134%). The prevalences of hypertension and type 2 diabetes mellitus are also higher in Reunion Island compared to mainland France [3].

In addition, given its geographical position in the South-West Indian Ocean (SWIO) region and its proximity to the eastern coast of Africa, a hot spot for emerging infectious diseases, and increasing trends of positive Indian ocean dipole [4], Reunion Island suffers global warming and is particularly exposed to zoonotic and vector-borne diseases. For instance, the SWIO region was the theater of the first ever observed large-scale chikungunya epidemic in 2005–2006, which affected 300,000 people (38%) in Reunion Island alone [5]. Moreover, the island has been facing recurrent seasonal outbreaks of dengue [6, 7] and endemic zoonotic diseases are also very common with yearly case increases of leptospirosis and murine typhus [7, 8].

### Hypotheses

First, we hypothesize a causal pathway between past arboviral infections and subsequent cardiometabolic complications due to low persisting inflammation, which could pave the way of insulin resistance and atherothrombosis years after. This is currently supported at the acute and post-acute stage of both chikungunya and dengue [9–12] with an increased risk of major cardiovascular events (MACE) within two weeks post-acute infection, as in the Taiwanese Health Insurance database [13]. In the long term, dengue may increase carotid-intima media thickness in children an average 8 years after experiencing dengue hemorrhagic fever [14]. According to the initial findings of the REDIACHIK follow-up study, exposure to CHIKV may contribute to the transition from normoglycemia towards prediabetes, and to the progression from prediabetes to diabetes, while it might also increase the risk of MACE [15].

Moreover, variations in microbiota composition have been observed in obesity, diabetes and cardiometabolic diseases disease [16]. Studies have shown the role of periodontal microbiota in inducing a pro-inflammatory adipokine secretory profile and oxidative stress [17, 18] and periodontal disease has been established as an independent risk factor for coronary heart disease [19, 20]. We hypothesize that periodontal bacteria can reach adipocytes in obese patients and induce a pro-inflammatory profile that can participate in insulin resistance.

Mental health has been linked to cardiometabolic and infectious diseases. Domestic violence particularly violence towards women is frequent in Reunion Island [21]. These stressful life events, such as posttraumatic stress disorder (PTSD), are positively associated with the development of CVD [22–25]. Moreover, evidence suggests that the gut microbiota can make individuals more susceptible to develop PTSD after a traumatic event [26].

While large cohorts that could adress such questions exist in France, such as CONSTANCES [27], GAZEL [28] and E3N [29], none of these includes participants from overseas territories. Including individuals of theses regions could provide unique and valuable data, as well as reliable indicators to help decision-making. By creating a biobank that is unique in this part of the world, our primary objective is first to describe the prevalence of cardiometabolic and infectious diseases in Reunion Island. Our secondary objective is to explore the causal mechanisms linking these diseases and to identify their common and specific determinants relying on a holistic approach. Different health issues will be explored in these pathways, such as microbiota, anxiety and PTSD, subclinical bronchial obstruction, consumption of products from local biodiversity, cognitive functions, social health inequalities, and autonomic nervous system function. This will allow to draw a conceptual framework underpinning the links between these diseases and to identify their determinants that could be targeted for global health promotion purposes in the context of Reunion Island.

## Methods

### Study Design

The REUNION study is an ongoing prospective study promoted by INSERM (No. C19-68), that received institutional support by University of La Réunion and CHU of La Réunion. The study protocol was approved by the Ethics Committee (CPP No. 20.12.15.47129, Clinical Trial NCT05400824). This article describes methods of recruitment of participants and data collection at baseline. This population-based sample is then intended for long term prospective follow-up.

### Population Sampling

From January 2022, participants are being recruited from the general population and invited to a medical research center, Plateforme de Recherche Clinique et Translationnelle in Saint Pierre or in a mobile examination center, after signing an informed consent form. Eligible subjects for the study include men and women aged between 18 and 67 years who understand and read French. Participants are volunteers and do not receive any financial compensation.

The sample of eligible subjects invited to participate in the study is obtained according to two different methods.

- Firstly, polling lists (nominal lists for French inhabitants over the aged of 18 years) available at each town hall in the survey area (Saint Pierre district) were used for random selection of the general population. A two-stage stratified random sampling approach was used, with stratification based on sex and age (five 10-year age groups). We used a disproportionate sampling technique, as the strata were all of equal size (50% men, 50% women, and then 20% in each age group).
- Secondly, eligible participants were selected by random generation of cell phone numbers with area code limited to Reunion Island (0692 and 0693), provided they are the main user of the phone line for private purposes and reside in Réunion.

### Collected Questionnaires

Extensive questionnaires are completed by the participants with the help of trained medical staff. The questionnaires collect data on various factors including sociodemographic characteristics, education level, occupational activity, lifestyle, leisure time physical activity, nutrition and diet, exposure to vector-borne diseases, medical history, cardiovascular risk factors and current treatment. Cognitive assessment questionnaires including Mini Mental State Examination (MMSE) [30], Instrumental Activities of Daily Living (IADL) Scale [31], Grober–Buschke test [32], and Trail making test [33] are completed from age 45. The Hospital Anxiety and Depression (HAD) scale [34], Life Events Checklist (LEC-5) [35] and Post-traumatic Stress Disorder Checklist (PCL-5) for DSM-5 [36] are also completed. These tests have been validated and used in other French population cohorts [33, 37]. Various components of social inequalities in health are also assessed: perceived health, social isolation and level of education, geographical identification of places of residence and work and access to healthcare. Data on ethnic origins is collected according to a questionnaire that has already been used in international publications by a team of geneticists studying the ethnic origins of the Reunionese population and their representations [38].

### Clinical Measurements

Anthropometric measurements, including height, body weight, and waist circumferences, are conducted according to detailed standard operating procedures that have been developed to describe measurement method for each parameter in order to obtain high quality medical data. Blood pressure is measured on both arms, first after a five-minute rest and then after one minute. Body temperature is also recorded. The monofilament test is performed following international guidelines for diagnosing peripheral neuropathy. The ankle-brachial index (ABI) is measured using a standard blood pressure cuff and a handheld Doppler, according to standardized procedures. The Ewing tests [39] are carried out by a specially trained nurse under the supervision of a doctor. Three of these tests assess parasympathetic function, measuring heart rate responses to deep breathing, standing, and to the Valsalva maneuver. The other two tests assess sympathetic function, measuring blood pressure response to orthostatism and to a sustained handgrip.

### Biological Sample Collection

A nurse collects fasting venous blood (48 mL) for immediate routine blood tests and for delayed tests, following the guidelines of the Human Plasma Proteome Project (HUPO) [40]. After centrifugation, plasma is obtained and DNA is extracted from the buffy coat. Mid-stream urine samples and stimulated saliva samples are collected after chewing paraffin gum for five minutes. These samples are divided into aliquots and sent to a biobank for long-term storage at − 80 °C. For the analysis of certain pollutants (pesticides/endocrine disruptors) and heavy metals such as mercury, a lock of hair is sampled after a cord has been tied 1 cm from the scalp (for orientation). The hair sample is then stored at room temperature, protected from light.

### Microbiota Samples

Samples of oral microbiota (periodontal), skin microbiota (ante-cubital fossa) and feces were collected. All samples were immediately stored at -80 °C after collection at the Centre de Ressources Biologique of the CHU de La Réunion.

### Additional Tests

A standard 12-lead ECG is performed and interpreted independently by two trained doctors. Finger–toe pulse wave velocity, using pOpmetre® (Axelife SAS, France) to assess the pulse wave transit time between the finger and toe, is used to evaluate arterial stiffness [41]. Finger–toe pulse wave velocity using pOpmetre® (Axelife SAS, France) to assess the pulse wave transit time between the finger and the toe is used to evaluate arterial stiffness [41].Doppler ultrasound is carried out by a trained vascular physician using Samsung® HM70A with linear probe L4-7 to assess the carotid and femoral arteries. Intima-media thickness (IMT), resistive index, plaque characterization, and degree of stenosis are measured in a sub-sample of participants. The measurement of exhaled carbon monoxide and other biomarkers (such as hydroxy-butyrate) is analyzed using an electronic nose (Alpha-mos, Toulouse, France). Forced vital capacity and forced expiratory volume in one second (FEV1) are measured at rest using a Spirobank 2® spirometer by a specially trained nurse.

### Endpoints

Our primary objective is to estimate prevalence of cardiometabolic and infectious diseases in Reunion Island, defined as:

- the prevalence of diabetes based on fasting glucose levels ≥ 7.0 mmol/l [42], hypertension based on office measurement ≥ 140/90 mmHg [43], obesity based on body mass index ≥ 30 kg/m2, dyslipidemia [44], and tobacco consumption,
- the presence of markers of subclinical atherosclerosis: ABI, IMT, presence of carotid and femoral plaques, arterial stiffness [41], proteinuria, SCORE2 risk score [43],
- commercially available recombinant proteins derived from the envelope or non-structural proteins of chikungunya, dengue, West Nile, Japanese encephalitis, Ross River, Usutu viruses will be used in ELISA test as the first line of screening. Positive samples will then be further screened using more specific tests targeting the domain II or III envelope genes with Luminex technology, which has already been used successfully in high-throughput seroprevalence studies [45, 46]. Additionally, specific neutralizing antibody tests [47] can be conducted against a collection of arboviruses available in our BSL3 lab,
- sociological, cognitive and behavioral patterns analyzed through questionnaires related to transmissions of these infectious diseases.

Our secondary endpoints include the assessment of the following:

- composition of intestinal, periodontal and skin microbiota: alpha and beta diversity, as well as relative abundance at different taxonomic levels [48],
- bronchial obstruction: forced expiratory volume in one second (FEV1) and Tiffeneau-Pinelli index (FEV1/forced vital capacity) [49],
- diet characteristics: frequency and quantity of consumption of various substances from local biodiversity, sodium intake, alcohol consumption, physical exercise,
- mental health and cognitive functions: LEC-5 scale [35], PCL-5 scale [36], HAD scale [34], MMSE [30], Codes test, Trail making tests [33], IADL scale [31],
- social inequalities in health: assessment of social isolation and level of education, residence and work place, access to healthcare, results of a specific questionnaire concerning ethnic origin as described above [38],
- autonomic dysfunction: digestive or urinary symptoms, erectile dysfunction, pupillary areflexia, heart rate variability, Ewing’s tests [39], screening for diabetic neuropathy using a 10 g Semmes-Weinstein monofilament,
- innovative lipid biomarkers: LDL/HDL profile (particle size by electrophoresis and NMR) [50], Lp(a) concentration, number of kringle repeats, myeloperoxidase, circulating DNA, cytokine measurement by multiplex ELISA,
- seroprevalence of exposure to periodontal bacteria (specific antibodies to *Porphyromonas gingivalis*, *Treponema denticola*, *Tannerella forsythia*, *Aggregatibacter actinomycetemcomitans*) and arbovirus diseases [17].
- markers resulting from multi-omics approaches (particularly proteomics and metabolomics [50]),
- frequency of certain polymorphisms concerning target genes involved in cardiometabolic (apolipoproteins such as apolipoprotein E [51], enzymes involved in iron metabolism such as heme oxygenase or haptoglobin),
- polymorphism of the ADCY9 gene to explain the response to cholesteryl ester transfer protein (CETP) inhibitors (specifically Dalcetrapib that impact plasma HDL concentrations) in the context of pharmacogenomic studies [52],
- polymorphisms of genes involved in resistance to infection such as Interleukin 1 receptor antagonist variable number tandem-repeat (IL1RN VNTR) polymorphism in the case of chikungunya infection [53],
- susceptibility genes for metabolic syndrome and cardiovascular complications, as well as infectious diseases using unbiased approaches including genomics, metabolomics and proteomics and complex network and machine learning approaches [54],
- population exposure to mosquito and tick bites:
  - evaluation of serum antibody responses to salivary proteins/peptides specific to the Aedes albopictus mosquito, a major vector in Réunion. Specific candidates peptides, such as the Nterm-34 kDa-Aedes peptide, the Nterm-34 kDa-Albo, and 34 kDa-internal-Albo peptides, have been validated as biomarkers for Aedes albopictus bites with no cross-reactivity with other arthropods or other organisms [55, 56],
  - infectious history: in the case of dengue, consecutive infections with different serotypes can influence the severity of the disease through the phenomenon of facilitation called antibody-dependent enhancement [57],
  - population exposure to environments contaminated by Leptospira [58],
- seroprevalence of SARS-COV-2 infections.

### Sample size

Considering an expected dropout rate of 20% due to moving since last registration on electoral roll, a 30% rate of non-response, and a first order risk set at 0.05, a sample of 2,000 subjects will result in a 1.8% precision for an estimated prevalence of 22% (which is the expected prevalence of diabetes in Reunion Island) and in a 2.0% precision for an estimated prevalence of 38% (which is the expected prevalence of hypertension in Reunion Island). Half of subjects (1000) will be included through polling list method and the other 1000 by the random phone number method.

## Discussion

Cardiometabolic diseases and their risk factors seem to be more common in Reunion Island compared to mainland France. However, the currently available epidemiological data on cardio-metabolic diseases and their risk factors in Réunion are either incomplete or outdated. Moreover, Reunion Island’s unique mixed population of African, Indian, Chinese and European origin and the rapid epidemiological transition occurring in the island, highlight the need for establishing population-based prospective cohorts. Consequently, there is a clear demand for recent and up-to-date data that adhere to international definitions and recommendations.

Reunion Island is also particularly exposed to zoonotic and vector-borne diseases, including human leptospirosis, chikungunya and dengue. While the occurrence of infectious diseases can be explained by Réunion’s particular geographical location in the South-West Indian Ocean and its proximity to the east coast of Africa, the higher incidence of cardio-metabolic disease cannot be entirely explained by differences in healthcare provision, which is now broadly comparable to mainland France. This over-representation of cardio-metabolic diseases could be partly explained by pathophysiological mechanisms that are still poorly understood. Our hypotheses focus on the role of the microbiota (intestinal, oral or cutaneous) and its links with chronic inflammation, oxidative stress, co-infections of infectious agents and emerging and re-emerging pathogens. Several studies support these hypotheses: (i) an association between periodontal disease and CVD has already been reported in patients [19, 20] and periodontal bacteria have been observed in carotid samples of patients with diabetes [17], (ii) the skin microbiota is involved in differential attraction of *Anopheles* mosquitoes [59, 60] and (iii) an association between dengue outcome and lipid profile has already been reported, with lower LDL cholesterol levels in patients with severe outcomes [61–63].

This study is a collaborative, multidisciplinary, and cross-disciplinary project that focuses on the relationship between cardiometabolic and infectious pathologies, their environmental, genetic, biochemical and microbiological risk factors. The study also investigates the qualitative and quantitative composition of various microbiota and their interactions. In order to support the research teams and foster national and international collaborations, a biobank will be established to contain plasma, DNA urine samples as well as samples of oral, intestinal and skin microbiota. This collection will serve as a unique clinico-biological resource.

In summary, data from the ongoing REUNION study should provide new insights into the prevalence of cardiometabolic and infectious diseases, as well as their potential associations through the examination of various environmental pathways and a wide range of health aspects. The current study has several advantages, including its prospective design and the availability of a unique biobank, which will allow us to gather extensive data and better understand the specific health profiles of the Reunionese population. This will potentially uncover explanations for the over-representation of certain diseases and open up avenues for further exploration.

## Data Availability

No datasets were generated or analysed during the current study.
